# Anais Brasileiros de Dermatologia – Impact Factor and CiteScore for 2024^⋆^^⋆^

**DOI:** 10.1016/j.abd.2025.501168

**Published:** 2025-07-30

**Authors:** Sílvio Alencar Marques, Ana Maria Roselino, Hiram Larangeira de Almeida Junior, Luciana Patrícia Fernandes Abbade

**Affiliations:** a Department of Infectology, Dermatology, Diagnostic Imaging and Radiotherapy, Faculdade de Medicina, Universidade Estadual Paulista, Botucatu, SP, Brazil; b Department of Internal Medicine, Division of Dermatology, Faculdade de Medicina de Ribeirão Preto, Universidade de São Paulo, Ribeirão Preto, SP, Brazil; c Department of Dermatology, Universidade Federal de Pelotas, Pelotas, RS, Brazil; d Department of Dermatology, Universidade Católica de Pelotas, Pelotas, RS, Brazil

Dear Colleagues,

Recently, on June 18, 2025, the Journal of Citation Reports released the metrics for 2024 of the 96 audited journals in Dermatology and related specialties. The various metrics calculated are intended to show the performance of the journals and define, even if this is not an explicit intention, and there may be biases, their ranking in their respective areas of activity or specialty. And once again, we are happy to be able to communicate to the national and international dermatology community the progress of the Impact Factor (IF), and items contributing to the composition of the IF and CiteScore of the *Anais Brasileiros de Dermatologia* (ABD). It is important to emphasize that we understand this as a collective achievement, the result of the efforts of the body of Scientific and Technical Editors, the contributions of countless Authors, and the quality of the voluntary work of the Reviewers. Although the metrics are somewhat distinct in their calculation methodologies, the different metrics allow several inferences and are indicative of paths to be maintained and others to be corrected and improved.

The IF of ABD for 2024 increased to 3.6; one point above the IF for 2023.^1^ This progress places us in 18th place among the 96 evaluated international journals, therefore in the first quartile - Q1, and in the 3rd place among Brazilian medical journals. Resulting from practically constant growth since the indexing of ABD in PubMed/Medline in 2009, considering the values ​​of its first IF = 0.337 (2010). The CiteScore, which uses methodology and databases from different source journals, also increased, albeit from a modest 0.1 to 2.5. It is obvious that these advances, particularly in the IF, are very welcome, but they do not satisfy or content us, since the vigor and productivity of Ibero-Latin American dermatology qualify us for positions of greater prominence. Anyhow, such results justify the celebrations of ABD on its centenary.

The metrics and auxiliary data provided allow Editors to identify areas or subjects with the greatest impact and repercussion and, consequently, to expand, as much as possible, the space allocated to such topics. Likewise, to identify subjects or topics with less or no repercussion and impact. The ABD has historically been a journal with multiple subjects and of interest to readers, as it prioritizes the most classic clinical dermatology, allocating space to aesthetic and surgical dermatology, and also to tropical and infectious dermatology and dermatopathology. And, it uses the “Letters” resource with fewer words and figures, to expand the allocated space to portray the indisputable wealth of the clinical and therapeutic universe in dermatology.^2^, ^3^, ^4^, ^5^, ^6^

The numbers reported above suggest that we are on the right track: a journal with multiple sections, robust in its emphasis on clinical dermatology in its broadest sense, and that remains demanding in the intellectual, visual, and editorial quality of the articles. However, we want to go further, and in 2025, the ABD will start publishing 35 articles per issue, or 15% more, as an essential initiative to avoid a backlog of approved articles awaiting publication. Furthermore, what we believe to be of great importance in terms of increasing the visibility and equalization of articles: starting with volume 101 (1), the ABD will be published exclusively in English, both online and in print, without changing its historic and century-old name.^7^ Therefore, starting in September 2025, all articles will have to be submitted in English.

We will continue to count on the financial support and infrastructure of the Brazilian Society of Dermatology and on the quality of the Authors and Reviewers who honor us with their work, observations, and dissemination.

We would like to give our thanks to all of you.

## Financial support

None declared.

## Authors' contributions

Silvio Alencar Marques: Approval of the final version of the manuscript; drafting and editing of the manuscript.

Ana Maria Roselino: Approval of the final version of the manuscript; drafting and editing of the manuscript.

Hiram Larangeira de Almeida Junior: Approval of the final version of the manuscript; drafting and editing of the manuscript.

Luciana P. Fernandes Abbade: Approval of the final version of the manuscript; drafting and editing of the manuscript.

## Conflicts of interest

None declared.
